# An adaptive Kalman filter approach for cardiorespiratory signal extraction and fusion of non-contacting sensors

**DOI:** 10.1186/1472-6947-14-37

**Published:** 2014-05-09

**Authors:** Jerome Foussier, Daniel Teichmann, Jing Jia, Berno Misgeld, Steffen Leonhardt

**Affiliations:** 1 Philips Chair for Medical Information Technology, Aachen University, Pauwelsstraße 20, 52074 Aachen, Germany; 2 Philips Medizin Systeme Böblingen GmbH, Hewlett-Packard-Straße 2, 71034 Böblingen, Germany

**Keywords:** Adaptive Kalman filter, Sensor fusion, Heart rate, Breathing rate, Signal processing

## Abstract

**Background:**

Extracting cardiorespiratory signals from non-invasive and non-contacting sensor arrangements, i.e. magnetic induction sensors, is a challenging task. The respiratory and cardiac signals are mixed on top of a large and time-varying offset and are likely to be disturbed by measurement noise. Basic filtering techniques fail to extract relevant information for monitoring purposes.

**Methods:**

We present a real-time filtering system based on an adaptive Kalman filter approach that separates signal offsets, respiratory and heart signals from three different sensor channels. It continuously estimates respiration and heart rates, which are fed back into the system model to enhance performance. Sensor and system noise covariance matrices are automatically adapted to the aimed application, thus improving the signal separation capabilities. We apply the filtering to two different subjects with different heart rates and sensor properties and compare the results to the non-adaptive version of the same Kalman filter. Also, the performance, depending on the initialization of the filters, is analyzed using three different configurations ranging from best to worst case.

**Results:**

Extracted data are compared with reference heart rates derived from a standard pulse-photoplethysmographic sensor and respiration rates from a flowmeter. In the worst case for one of the subjects the adaptive filter obtains mean errors (standard deviations) of -0.2 min ^−1^ (0.3 min ^−1^) and -0.7 bpm (1.7 bpm) (compared to -0.2 min ^−1^ (0.4 min ^−1^) and 42.0 bpm (6.1 bpm) for the non-adaptive filter) for respiration and heart rate, respectively. In bad conditions the heart rate is only correctly measurable when the Kalman matrices are adapted to the target sensor signals. Also, the reduced mean error between the extracted offset and the raw sensor signal shows that adapting the Kalman filter continuously improves the ability to separate the desired signals from the raw sensor data. The average total computational time needed for the Kalman filters is under 25% of the total signal length rendering it possible to perform the filtering in real-time.

**Conclusions:**

It is possible to measure in real-time heart and breathing rates using an adaptive Kalman filter approach. Adapting the Kalman filter matrices improves the estimation results and makes the filter universally deployable when measuring cardiorespiratory signals.

## Background

The increase in both life quality expectancy and quality of life, together with improvements in medical support, has led to an increase in the mean age of the population in developed lands. The American Administration on Aging (AoA) predicts that, compared with the year 2000, the absolute number of people aged >65 years will double by 2030 and represent 19% of the total U.S. population [1]. The increase in the elderly population will lead to additional strains on the health-care system. Therefore, there is an explicit need to transfer clinical measurement devices and technology to the patient’s home. The goal of personal health care is to relieve clinicians and the clinical infrastructure by means of technical improvements, but without reduction in diagnostic and rehabilitation performance, e.g. with telemonitoring at home [2]. This implies that new technology needs to be integrated into daily activity which, compared with a well-defined clinical environment, poses considerable challenges in terms of signal acquisition and processing. More complex processing algorithms are needed to overcome noise, artifacts and multi-sensor problems. One algorithmic approach is the use of the Kalman filtering technique.

Since its introduction in the 1960s, the Kalman filter has become a well accepted and state-of-the-art approach for many applications, especially in the technical domain. Initially, computational capacity was limited and costly, making it difficult to use Kalman filters in real-time applications. However, over the years computational power has increased, also outside the personal computer domain. Today, high performance devices are available, including microcontrollers, digital signal processors or specialized computational units such as Field Programmable Gate Arrays (FPGA). Therefore, it is now possible to move complex mathematical computations into such devices working in a self-sufficient way. Many examples of technical applications using Kalman filters in real-time have been described [3,4]. In bio-medical engineering, Kalman filters are often applied to smooth or extract physiological signals, such as respiration and cardiac activity [5,6]. Also in the domain of Electrical Impedance Tomography (EIT), the Kalman filter is able to track fast changes in impedance [7]. The Kalman filter is adequate for the present work, as it is possible to integrate prior knowledge (e.g. about sensor or system noise or state transitions). In addition, the Kalman filter is capable to denoise, separate signals or fuse sensor data, all in one architecture. Compared to other filtering or signal separation methods the Kalman filter is able to perform in real-time with very little systemic delays.

To record evaluation data a non-contact measurement technique, called magnetic induction monitoring, was chosen. This technique is a relevant method since it comprises a variety of problems typical for non-contact monitoring of vital signs. The complete employed measurement setup is described in Section ‘Sensors and measurement setup’. Afterwards, the detailed working principle and the implementation of the Kalman filter is explained (Section ‘Kalman filter’). Then, heart and respiration rate extraction results of two different subjects are shown and discussed in Section ‘Results and discussion’. We oppose the non-adaptive, described in earlier work, to the developed adaptive Kalman filter and show the performance increase, especially when no a priori knowledge about the sensor system is present. Finally, the conclusions summarize the results and gives an outlook to future work (see Section ‘Conclusions’).

## Methods

### Sensors and measurement setup

The signal were acquired in voluntary self-experiments of the first two authors (JF and DT). Both signed an informed consent to participate in this study. The local ethics committee decided that this type of study with this device was not in the scope of their responsibility (internal reference number EK 013/14). In addition it was stated that the self-measured data can be used for publication. Both signed an informed consent to participate in this study. Consider, that this work is not conceived to be an extensive study but rather a proof of concept. Magnetic induction monitoring is a non-contact technique for recording thoracic activity. The technique is based on magnetic coupling between the thorax and a nearby sensor-coil, as developed by Teichmann *et al.*[8,9]. As the coil has no conductive contact with the skin it is called a non-contact technique. The sensor-coil is driven by an alternating current and sends out an alternating magnetic field. This field induces eddy currents within conductive objects in the vicinity of the coil. In turn, these eddy currents reinduce a secondary alternating magnetic field, which affects the primary one and thereby changes the reflective impedance of the coil. If the coil is placed near the thorax, cardiorespiratory activity modulates the impedance of the coil due to motions of inner organs and the thoracic wall and/or because of changes in conductivity, e.g. caused by more or less air in the lungs or blood shifts in the heart. In this way, respiration and pulse can be easily obtained by measuring the impedance changes of the sensor-coil.

**Figure 1:**
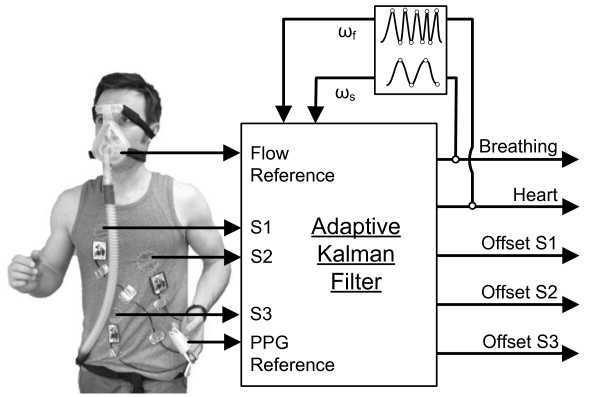
**Kalman filter sensor fusion.** Kalman filter sensor fusion (sensors: S1 to S3 with flow and PPG references) and vital signs extraction with frequency adaptation.

In this work, processed data were collected by three magnetic induction sensors attached to the thorax, as described in [10,11]. The sensors were located on left breast, right breast and stomach level (Figure 1). With this setting it is possible to detect motion patterns. In addition to those three sensor signals, two reference channels acquire data in parallel: one for a pulse photoplethysmographic (PPG) finger clip sensor (ChipOx, Corscience GmbH & Co. KG) the other for a thermal mass flowmeter (Model 4040, TSI). ECG and respiratory induction plethysmography (RIP) as references have been discarded as they might influence the magnetic measurement system. All measurements have been performed in resting state, so that no artifacts due to running or other movements are induced since we want to show the performance of being able to extract heart and respiration rates. The task of detecting artifacts is not part of this work, since motion patterns have been analyzed in previous work [11]. Magnetic induction monitoring is completely contact free, i.e. no mechanical, conductive or optical contact is necessary. Although this offers a considerable advantage over other monitoring methods, there are problems related to this and other non-contact monitoring techniques. A major drawback of magnetic induction monitoring is its sensitivity to other thoracic motion not related to respiration or pulse; therefore, motion artifacts are a common problem. Also, the signal content related to respiration is much higher than the cardiac-related signal content, since the thoracic conductivity distribution is more strongly affected by respiratory motion. Often, the higher harmonics of the respiratory signal may overlap the much smaller cardiac signal; in this case signal separation by common frequency filtering is not possible. To overcome these issues (at least to some extent), an adaptive Kalman filter was developed, based on previous work [5,12]. The Kalman filter system consists of three individual sensor signals (S1 to S3), that are direct inputs of the Kalman filter (Figure 1). In addition to the filter system described earlier [12], the respiratory rate and heart rate are estimated continuously and fed back to the Kalman filter where internal states and matrices are updated. Also some of the state matrices containing information about the measurement system are updated recursively and thus, automatically adapting itself to any new configuration. This procedure is described in detail in the following sections.

**Figure 2:**
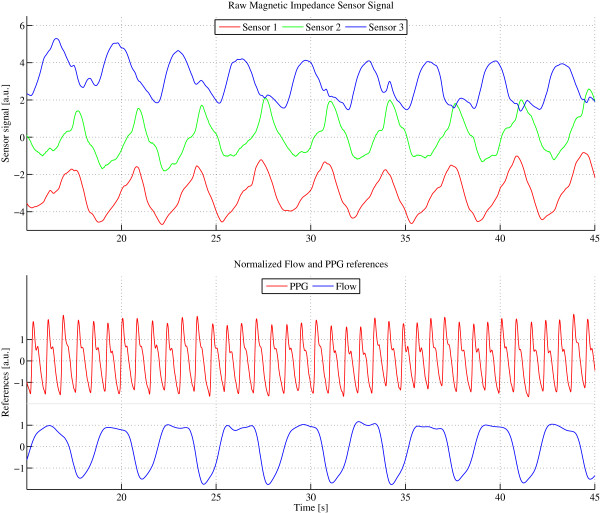
**Raw signal example with parallel Flow and PPG reference recordings.** Raw signal of sensor 1-3 example (top with arbitrary units [a.u.]) with parallel flow and PPG reference recordings (bottom with arbitrary units [a.u.]) (30 s window).

The raw sensor data were acquired at a sampling rate of 95 Hz, which is also the processing rate of the Kalman filter. No pre-processing of the data is performed. Figure 2 presents a 30-s representative example of the employed sensor signal with the PPG and flow reference acquired in parallel.

In summary, the Kalman filter has to deal with the following signal properties: 

● A very high offset compared to the signal amplitude

● Respiration is visible in the time domain

● Heart activity is only slightly visible in the time domain (very low ratio of heart to respiration signal level)

● Good signal-to-noise ratio (SNR) for the respiratory signal, since noise is not noticeable in the time domain for this sensor

● Higher harmonics of the respiratory signal may overlap the pulse signal in the frequency domain

### Kalman filter

In 1960, R.E. Kalman reported a new method for linear filtering and solving problems related to prediction [13]. Generally, the so-called “Kalman filter” consists of mathematical equations that represent an efficient way to predict a future and/or unknown state of a system, based only on the use of the preceding step. The calculations are very efficient so that they can be performed and implemented in today’s standard default personal computers, in digital signal processors (DSP) and in microcontrollers, to work in real-time applications. When process and sensor noise have time-dependent characteristics, the filter is also called a “time-varying Kalman filter”. The filter finds a prediction value $\hat{x}$ with minimum variance for a disturbed state vector **x**[14]. The transitions from one state to another are represented in the state transition matrix **A** when no disturbance is present.

System and measurement noise, which are assumed to be white (zero mean) and statistically independent from each other, are represented by **w** and **v**, with their co-variance matrices **Q** and **R** (both hermitian symmetric and positive semidefinite), respectively. External disturbances (e.g. systematic errors) are fed into the system with the control vector **u** and the matrix **B**, that describe the dynamics of the disturbance. Finally, the measurement matrix **H** describes the integration of a real measurement into the filter procedure.

The matrices **A**, **Q**, **R**, **B** and **H** are generally time invariant, but may be adaptable over time. Especially when the acquired quality of the sensor data changes over time, the values of **Q**, **R** and **H** should take this into consideration. When the assumed system model is exposed to changes, **A** and **B** also need to be updated. All time variant matrices and vectors are denoted with a small index *k*.

(1) $$\begin{array}{l} {\hat{x}}_{k}^{-}=A_{k}{\hat{x}}_{k-1}+B_{k}u_{k-1} \end{array}$$

(2) $$\begin{array}{l} P_{k}^{-}=A_{k}P_{k-1}{A_{k}}^{T}+Q_{k} \end{array}$$

(3) $$\begin{array}{l} K_{k}=P_{k}^{-}{H_{k}}^{T}{\left(H_{k}P_{k}^{-}{H_{k}}^{T}+R_{k}\right)}^{-1} \end{array}$$

(4) $$\begin{array}{l} {\hat{x}}_{k}={\hat{x}}_{k}^{-}+K_{k}\left(z_{k}-H_{k}{\hat{x}}_{k}^{-}\right) \end{array}$$

(5) $$\begin{array}{l} P_{k}=\left(I-K_{k}H_{k}\right)P_{k}^{-} \end{array}$$

The prediction step, also called “time update”, is described with (1) and (2). The aim of this step is to minimize the co-variance of the estimation error which represents a degree of uncertainty of the estimation [14]. Note that both equations are only valid for *k*>0. The initial values of ${\hat{x}}_{0}$ and *P*_0_ have to be determined before the first iteration. The control input *u*_*k*_ is not used in this model and is set to the zero vector, thus the matrix *B*_*k*_ in (1) can be ignored.

The Kalman gain *K*_*k*_ defined in (3) weights the innovation with respect to the measurement error co-variance matrix *R*_*k*_ and the estimation error co-variance matrix $P_{k}^{-}$ directly related to *Q*_*k*_. Higher values in *R*_*k*_ give more importance to the real measurement, whereas higher values for *Q*_*k*_ put more trust in the estimation of a state. The choice of *R*_*k*_ and *Q*_*k*_ is crucial for optimal filter performance. The correction step, also called “measurement update”, integrates the innovation of a new measurement *z*_*k*_ to the estimated measurement in (4). Finally, the co-variance matrix of the estimation error *P*_*k*_ is updatedin (5).

The advantage of a Kalman filter is that it does not act as a pure filter, but also signal separation and fusion are realizable in a single implementation. Signal separation is done by defining several system states that are desired as separate outputs of the filter. Fusion is realized by expanding the measurement matrix **H** or *H*_*k*_ to multiple sensors. With this, the Kalman technique is applicable to a wide field of applications, especially where no exact system model is known [14].

#### Signal extraction and separation

The employed system was developed to work with three sensors, each measuring heart and breathing activity separately on top of a large offset (Figure 2). Therefore, the Kalman filter was split into seven states, two for heart activity (named *X*_*f*,*k*_ and *V*_*f*,*k*_), two for respiration (named *X*_*s*,*k*_ and *V*_*s*,*k*_) and three sensor offset estimates (named *C*_1,*k*_, *C*_2,*k*_ and *C*_3,*k*_), merged into one estimated state vector ${\hat{x}}_{k}$:

(6) $${\hat{x}}_{k}={\left(\begin{array}{c} X_{f,k},V_{f,k},X_{s,k},V_{s,k},C_{1,k},C_{2,k},C_{3,k} \end{array}\right)}^{T},$$

In previous work, the state transition matrix **A** was time invariant [12]. It was designed to extract two sinusoidal-shaped signals (with different but fixed frequencies) and three offsets from three measurement sensor signals. It has been shown that the filter model for periodic motion is able to compensate (to some extent) for incorrect assumptions of the angular frequencies *ω*_*f*_ and *ω*_*s*_[5]. In conditions where either the real breathing frequency or the heart rate is different from the model assumption, the estimated states tend to measure wrong frequencies. Therefore, the model needs to be updated over time by feeding back the estimated frequency into an adaptive state transition matrix which has been derived from the work of Spincemaille *et al.*[5]:

(7) $$A_{k}=\left(\begin{array}{ccccccc} 1 & \mathrm{\Delta t} & 0 & 0 & 0 & 0 & 0\\ -\omega_{f,k}^{2}\mathrm{\Delta t} & 1 & 0 & 0 & 0 & 0 & 0\\ 0 & 0 & 1 & \mathrm{\Delta t} & 0 & 0 & 0\\ 0 & 0 & -\omega_{s,k}^{2}\mathrm{\Delta t} & 1 & 0 & 0 & 0\\ 0 & 0 & 0 & 0 & 1 & 0 & 0\\ 0 & 0 & 0 & 0 & 0 & 1 & 0\\ 0 & 0 & 0 & 0 & 0 & 0 & 1 \end{array}\right)$$

with *ω*_*s*,*k*_=2*π*·*f*_*s*,*k*_ and *ω*_*f*,*k*_=2*π*·*f*_*f*,*k*_ being the time changing angular frequencies of the breathing frequency *f*_*s*,*k*_ and the heart beat rate *f*_*f*,*k*_ at time step *k*, respectively.

The frequency measurement is based on a simple peak detector which extracts the location of maxima and minima in the signal and then computes the mean interval length between maximal and minimal points. The main advantage of a peak detector is the reduced computational complexity compared to other frequency measurement methods, e.g. based on the spectrum analysis. To accurately measure respiration and heart rate, the algorithm needs to buffer the output signal of the Kalman filter, where the denoted “Breathing” and “Heart” feedbacks in Figure 1 are represented by the internal Kalman states *X*_*s*,*k*_ and *X*_*f*,*k*_, respectively. Note that it is not possible to estimate the heart rate directly from the raw sensor signal, due to the small heart signal amplitude and a possible frequency overlap of respiratory harmonics. A buffer of 20 s (approx. 1900 samples) for respiration and of 10 s (approx. 950 samples) for the heart signal were chosen to guarantee at least two consecutive respiratory cycles and several heart beats within the buffer. However, having such a large buffer causes systematic delays in the frequency estimation of half of the buffer length, i.e. 10 s and 5 s, respectively. Generally, because heart and respiration rates change slowly, we do not need to adapt the system model at 95 Hz. In fact, only at every tenth sample a frequency measurement is performed, resulting in an effective estimation rate of 9.5 Hz reducing computational effort by 90%. The remaining nine samples are filled with the last valid estimation to keep the same sampling rate of 95 Hz as the rest of the Kalman filter. This high sampling rate is needed to keep systematic errors at a minimum. At 95 Hz, the maximum absolute time resolution to measure the time between two signal peaks is $\frac{1}{95\;\text{Hz}}=10.5\;\text{ms}$, inducing a measurement error increasing with the heart rate with 0.0175% per beat per minute (bpm) (e.g. at 80 bpm the relative error is up to 1.4% equivalent to an absolute error of 1.12 bpm). The frequency measurement procedure has been validated with simulated sinusoidal signals covering heart rates from 60 bpm to 120 bpm and respiration rates from 12 min−1 to 15 min−1.

For stability reasons, abrupt changes in the frequency measurement need to be avoided, e.g. false estimations of the frequencies or artifacts, directly fed into the state transition matrix *A*_*k*_ of the adaptive Kalman filter. Therefore, the estimated frequencies representing the heart and the respiration rates are each lowpass filtered with first order infinite impulse response (IIR) Butterworth filters, designed with the Matlab R2011a (MathWorks) “Signal Processing Toolbox” with cutoff frequencies of 0.1 Hz and 0.05 Hz, respectively.

In our previous work [12], the measurement noise co-variance matrix **R** was also time-invariant, fixed to the standard deviation of each sensor signal determined before the real measurement. Therefore, no continuous adaptation to changes in the sensor signal quality was possible. Since such adaptation is important for optimal performance and, generally, this type of a priori calibration is inconvenient for our work, the measurement noise was also estimated leading to a time variant matrix *R*_*k*_:

(8) $$R_{k}=\left(\begin{array}{ccc} 1 & 0 & 0\\ 0 & 1 & 0\\ 0 & 0 & 1 \end{array}\right)\cdot \sigma_{\text{noise},k}^{2},$$

with $\sigma_{\text{noise},k}={\left(\begin{array}{ccc} \sigma_{\text{noise},k}^{1} & \sigma_{\text{noise},k}^{2} & \sigma_{\text{noise},k}^{3} \end{array}\right)}^{T}$ as ideal measurement variances of sensor 1-3 at time step *k*, respectively. Generally, the ideal value is unknown and different for each sensor. So, at each discrete time step *k*, the noise co-variance matrix *R*_*k*_ has to be updated by estimating the standard deviation (std) of a numerically differentiated (diff) short signal segment. In our case the last 0.5 s (approx. 48 samples) *s*_*i*,*Δ*_ of each sensor *i* have been taken into account:

(9) $${\hat{\sigma }}_{\text{noise},k}^{i}=\frac{\mathrm{std}\left(\mathrm{diff}\left(s_{i,\Delta }\right)\right)}{\sqrt{2}}.$$

Note that all estimations that are described in this work have been computed every tenth sample and lowpass filtered in the same way as the heart rate estimation value as described previously (first order Butterworth filter with 0.1 Hz cutoff frequency). Also, the development of all the described estimations first has been applied to simulated signals, where noise and standard deviations of the heart and respiratory signals were known.

#### Signal filtering

Spincemaille *et al.* have shown [5] that same ratios of *R*_*k*_ and *Q*_*k*_ also give similar filtering performances. So, changing the *R*_*k*_ matrix also means adapting the system noise co-variance matrix *Q*_*k*_ appropriately:

(10) $$\;Q_{k}\;=\;\left(\;\begin{array}{ccccccc} 1 & 0 & 0 & 0 & 0 & 0 & 0\\ 0 & \omega_{f,k}^{2} & 0 & 0 & 0 & 0 & 0\\ 0 & 0 & 1 & 0 & 0 & 0 & 0\\ 0 & 0 & 0 & \omega_{s,k}^{2} & 0 & 0 & 0\\ 0 & 0 & 0 & 0 & {\left(\sigma_{\text{trend},k}^{1}\right)}^{2} & 0 & 0\\ 0 & 0 & 0 & 0 & 0 & {\left(\sigma_{\text{trend},k}^{2}\right)}^{2} & 0\\ 0 & 0 & 0 & 0 & 0 & 0 & {\left(\sigma_{\text{trend},k}^{3}\right)}^{2} \end{array}\;\right),$$

with variances of the long-term trend $\sigma_{\mathrm{trend},k}={\left(\sigma_{\text{trend},k}^{1}\;\sigma_{\text{trend},k}^{2}\;\sigma_{\text{trend},k}^{3}\right)}^{T}$ of sensors 1-3.

The co-variance matrix *Q*_0_ is initialized with the heart and breathing frequencies *ω*_*f*,*k*_ and *ω*_*s*,*k*_ and the trend variations $\sigma_{\text{trend},k}^{i}$ initialized by the user at time step *k*=0, respectively. We will show, that those values do not need to be very accurate, since they are automatically estimated over time. However, as the exact trend variance is unknown it is also estimated using the raw sensor signal:

(11) $${\left({\hat{\sigma }}_{\text{trend},k}^{i}\right)}^{2}=0.01\cdot {\left({\hat{\sigma }}_{\text{resp},k}^{i}\right)}^{2}$$

(12) $${\hat{\sigma }}_{\text{resp},k}^{i}=\mathrm{std}\left(l_{i,\Delta }\right),$$

with ${\hat{\sigma }}_{\text{resp},k}^{i}$ as standard deviation estimation of the respiration using the long-term segment *l*_*i*,*Δ*_ over the last 20 s (approx. 1900 samples). We defined that the trend variation corresponds to 1% of the respiratory variation. Lower entries in the matrix (10) mean that more trust is placed on the predicted model states than on the raw measurement. Higher frequencies also cause higher amplitudes in the differentiated Kalman state vector signals *V*_*f*,*k*_ and *V*_*s*,*k*_ and are proportional to $\omega_{f,k}^{2}$ and $\omega_{s,k}^{2}$, respectively. A high long-term variance in the sensor signal is assumed to be caused by respiration and heart beat. The values for the states *X*_*s*,*k*_ and *X*_*f*,*k*_ defined in eq. (6) normally are smaller than for *V*_*s*,*k*_ and *V*_*f*,*k*_. With this, more trust is given to the system model than to the measurement resulting in smoothed sinusoidal-shaped signals with angular frequencies of around *ω*_*s*,*k*_ and *ω*_*f*,*k*_, respectively. This simplifies the detection of peaks in the signal and improves frequency estimation. In fact, the states *V*_*s*,*k*_ and *V*_*f*,*k*_ contain more actual sensor information, thus more noise and possible artifacts are present; these states would need a processing step before being used for frequency estimation.

#### Sensor fusion

In general, we assumed that the sensor configuration is able to measure vital signs. The first four columns of the measurement matrix *H*_*k*_, representing the heart and respiration states of the estimated state vector ${\hat{x}}_{k}$, defined in (6), are updated by using the noise estimation previously described in (9), (12) and (15):

(13) $$h_{f,k}^{i}={\left(\frac{{\hat{\sigma }}_{\text{heart},k}^{i}}{{\hat{\sigma }}_{\text{noise},k}^{i}}\right)}^{2}\cdot \text{sca}l_{f}^{i}$$

(14) $$h_{s,k}^{i}={\left({\hat{\sigma }}_{\text{resp},k}^{i}\right)}^{2}\cdot \text{sca}l_{s}^{i}$$

(15) $${\hat{\sigma }}_{\text{heart},k}=\frac{\mathrm{std}\left(s_{i,\Delta }\right)}{\sqrt{2}},$$

with $\text{sca}l_{f}^{i}$ and $\text{sca}l_{s}^{i}$ as sensor specific scaling factors for measuring heart and respiratory activity. This is the only a priori information that has to be defined by the user. Generally, the general performance of the filter is not very sensitive to this scaling as the Kalman filter itself adapts to the average of all three sensors. Nevertheless, it might enhance the filtering performance. In our case we know that sensor 1 mainly contains cardiac information since it is attached right above the heart (see Figure 1) and all other sensors are located far away from the heart. Those are scaled to 10% as well as the respiratory data content of sensor 1:

(16) $$\mathrm{sca}l_{f}={\left(\text{sca}l_{f}^{1}\;\text{sca}l_{f}^{2}\;\text{sca}l_{f}^{3}\right)}^{T}={\left(1\;0.1\;0.1\right)}^{T}$$

(17) $$\text{sca}l_{f}={\left(\text{sca}l_{f}^{1}\;\text{sca}l_{f}^{2}\;\text{sca}l_{f}^{3}\right)}^{T}={\left(0.1\;1\;1\right)}^{T}.$$

If no a priori information is given, e.g. when the sensor location is unknown, the *scal*_*f*_ and *scal*_*s*_ settings should be chosen as (1 1 1)^*T*^ to give the same scaling to all sensors.

The last three columns in *H*_*k*_ indicate which offset in the estimated state vector ${\hat{x}}_{k}$ is linked to a specific sensor, finally leading to:

(18) $$H_{k}=\left(\begin{array}{lllllll} h_{f,k}^{1} & 0 & h_{s,k}^{1} & 0 & 1 & 0 & 0\\ h_{f,k}^{2} & 0 & h_{s,k}^{2} & 0 & 0 & 1 & 0\\ h_{f,k}^{3} & 0 & h_{s,k}^{3} & 0 & 0 & 0 & 1 \end{array}\right).$$

However, the offset links cannot be changed as they act totally independently. In (3) and (4) the sensor fusion is performed, as all measurement channels are joined into the estimated state vector ${\hat{x}}_{k}$ with the Kalman Gain *K*_*k*_.

## Results and discussion

In this section, the performance of the non-adaptive and the adaptive Kalman filter is evaluated by comparing the estimated respiration rates and heart rates with the reference flow and PPG signals. Instead of the peak detection and filtering methods described in Section ‘Signal extraction and separation’ we applied a spectrum based frequency estimator to the reference flow and PPG signals to obtain much higher frequency resolution. This estimator is not adequate for real-time application since it is computationally much more complex. Note that in the first 22.5 s (i.e. the minimal length of the buffers used for the respiration frequency and signal variance estimations), frequency estimation neither for the respiration rate nor for the heart rate is computed. Especially for the adaptive Kalman filter, it is important to regulate the frequencies in the model only when both frequencies are really measurable thus only when the Kalman filter is settled. This avoids the filter reaching an irreversible incorrect state. Therefore, during further analysis, the first 22.5 s are ignored as generally no valid and comparable data are available.

For the performance analysis between the adaptive and non-adaptive Kalman filter, we defined three different settings: 

● **SE**: When the *Sensor Estimation* settings is used, the whole raw sensor signal is first analyzed, giving the optimal estimations of variances. Note that this setting only works in a post-processing way, but it is used to create the best-case setting in this work.

● **DS**: The *Default Settings* should adequately work in most cases.

● **BS**: *Bad Settings* that give a very inappropriate definition of the system - the worst-case scenario when the user misconfigures the Kalman filter.

In Table 1 the initialization parameters for each setting and subject are shown. Note that for BS, especially the noise variances have been set to very high and the respiration/heart variances to relatively low values. This setting reflects a possible misconfiguration of the Kalman filter, e.g. when wrong a priori information are set by the user. With BS we want to demonstrate that the user does not have to care about the correct settings when using the adaptive Kalman filter contrarily to the non-adaptive filter. The SE setting, so the optimal settings determined by preprocessing the raw acquisition data, shows that the sensor amplitudes between both subjects are very different making it very difficult to estimate correct parameters from case to case. The ratios between the estimated heart and respiration deviation $\frac{{\hat{\sigma }}_{\text{heart},0}^{i}}{{\hat{\sigma }}_{\text{resp},0}^{i}}$ confirm the fact that sensor 1 contains most of the heart signal information with respect to the respiratory signal compared to all other sensors.

**Table 1:** Initialization parameters using different settings for subject 1 and 2

	**SE**	**SE**	**DS**	**DS**	**BS**	**BS**
**Subject**	1	2	1	2	1	2
***f***_***f***_**[Hz]**	1.3	1.7	1.5	1.5	1.0	1.0
***f***_***s***_**[Hz]**	0.3	0.2	0.1	0.1	0.1	0.1
${\hat{\sigma }}_{\text{trend},0}^{1}$	989.0	54.1	100.0	100.0	1.0	1.0
${\hat{\sigma }}_{\text{trend},0}^{2}$	159.2	819.9	100.0	100.0	1.0	1.0
${\hat{\sigma }}_{\text{trend},0}^{3}$	1057.7	1801.5	100.0	100.0	1.0	1.0
${\hat{\sigma }}_{\text{noise},0}^{1}$	53.6	6.7	10.0	10.0	1000.0	1000.0
${\hat{\sigma }}_{\text{noise},0}^{2}$	11.2	12.2	10.0	10.0	1000.0	1000.0
${\hat{\sigma }}_{\text{noise},0}^{3}$	64.2	15.6	10.0	10.0	1000.0	1000.0
${\hat{\sigma }}_{\text{heart},0}^{1}$	1112.1	70.1	100.0	100.0	1.0	1.0
${\hat{\sigma }}_{\text{heart},0}^{2}$	195.8	198.8	100.0	100.0	1.0	1.0
${\hat{\sigma }}_{\text{heart},0}^{3}$	1605.8	454.8	100.0	100.0	1.0	1.0
${\hat{\sigma }}_{\text{resp},0}^{1}$	10195.7	191.2	10000.0	10000.0	1.0	1.0
${\hat{\sigma }}_{\text{resp},0}^{2}$	1114.4	4137.1	10000.0	10000.0	1.0	1.0
${\hat{\sigma }}_{\text{resp},0}^{3}$	8498.0	5846.5	10000.0	10000.0	1.0	1.0
$\text{sca}l_{f}^{1}$	1.0	1.0	1.0	1.0	1.0	1.0
$\text{sca}l_{f}^{2}$	0.1	0.1	0.1	0.1	0.1	0.1
$\text{sca}l_{f}^{3}$	0.1	0.1	0.1	0.1	0.1	0.1
$\text{sca}l_{s}^{1}$	0.1	0.1	0.1	0.1	0.1	0.1
$\text{sca}l_{s}^{2}$	1.0	1.0	1.0	1.0	1.0	1.0
$\text{sca}l_{s}^{3}$	1.0	1.0	1.0	1.0	1.0	1.0

**Figure 3:**
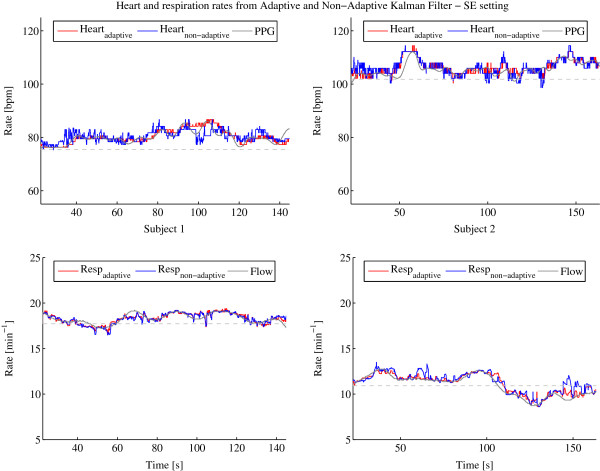
**Heart and respiration rates determined by adaptive and non-adaptive Kalman filter with PPG and flow reference using the SE setting.** Heart (top) and respiration (bottom) rates determined by adaptive (red) and non-adaptive (blue) Kalman filter with PPG and flow reference (gray) using the SE setting for subject 1 (left column) and 2 (right column). The dotted gray lines indicate the starting frequencies of the heart and respiration rates defined in the setting at startup.

**Figure 4:**
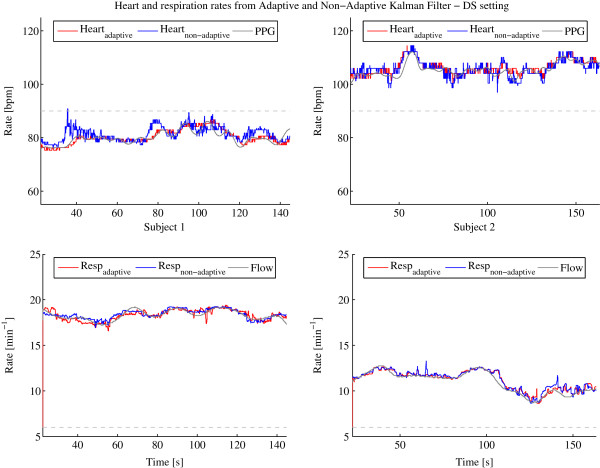
**Heart and respiration rates determined by adaptive and non-adaptive Kalman filter with PPG and flow reference using the DS setting.** Heart (top) and respiration (bottom) rates determined by adaptive (red) and non-adaptive (blue) Kalman filter with PPG and flow reference (gray) using the DS setting for subject 1 (left column) and 2 (right column). The dotted gray lines indicate the starting frequencies of the heart and respiration rates defined in the setting at startup.

**Figure 5:**
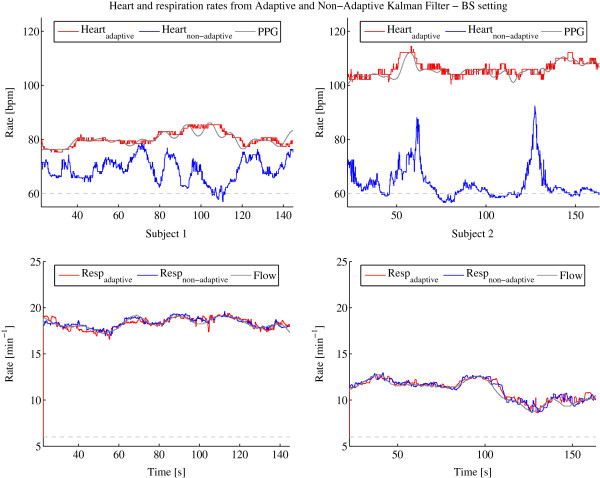
**Heart and respiration rates determined by adaptive and non-adaptive Kalman filter with PPG and flow reference using the BS setting.** Heart (top) and respiration (bottom) rates determined by adaptive (red) and non-adaptive (blue) Kalman filter with PPG and flow reference (gray) using the BS setting for subject 1 (left column) and 2 (right column). The dotted gray lines indicate the starting frequencies of the heart and respiration rates defined in the setting at startup.

The available signal segment lengths for subject 1 are 144.8 s and 163.4 s for subject 2. The results of the frequency estimations of heart and respiration over those segments are shown in Figure 3, Figure 4 and Figure 5 after having applied the procedures described in Section ‘Kalman filter’ with the adaptive and non-adaptive Kalman filter for the SE, DS and BS settings, respectively. The configurations SE and DS show very good frequency estimation performance for both Kalman filters, the adaptive and non-adaptive version, and for both subjects. The estimated rates follow the reference lines very well. To support the visual analysis, the mean difference and standard deviation between the reference and the estimated frequency have been computed which are listed for each setting and subject in Table 2. Settings SE and DS generate errors that are all in the same range, showing almost equal performances between both Kalman filters. The standard deviations are slightly smaller for the adaptive filter. The mean deviations are within the systematic error range resulting from the 95 Hz sampling rate as described in Section ‘Signal extraction and separation’. However, the BS setting gives a totally different result. The non-adaptive Kalman filter is not able to extract the heart rate any more but since the respiration signal is much higher in amplitude than the heart signal, even this bad configuration is usable for respiration rate estimation. The adaptive Kalman filter compensates the bad settings by adapting to the sensor recursively and allowing it to extract heart rate estimations much more accurately. Table 2 also clearly confirms this finding where the heart signal mean error for the adaptive Kalman filter is about sixty times and the standard deviation about four times smaller than for the non-adaptive Kalman filter.

**Table 2:** Mean Error (standard deviation) between flow/PPG reference and estimated respiration/heart rates for the non-adaptive and adaptive (Ad.) Kalman filter

	**Subj.**	**Resp.**	**Resp. Ad.**	**Heart**	**Heart Ad.**
		**[min**^**−1**^**]**	**[min**^**−1**^**]**	**[bpm]**	**[bpm]**
**SE**	1	0.0 (0.3)	0.0 (0.3)	-0.3 (2.1)	-0.2 (1.4)
**SE**	2	-0.3 (0.5)	-0.2 (0.3)	-0.8 (2.3)	-0.7 (1.7)
**DS**	1	-0.1 (0.3)	0.0 (0.3)	-1.2 (2.3)	-0.2 (1.5)
**DS**	2	-0.3 (0.4)	-0.2 (0.3)	-0.3 (2.2)	-0.8 (1.7)
**BS**	1	-0.1 (0.2)	0.0 (0.3)	11.2 (5.7)	-0.2 (1.5)
**BS**	2	-0.2 (0.4)	-0.2 (0.3)	42.0 (6.1)	-0.7 (1.7)

**Figure 6:**
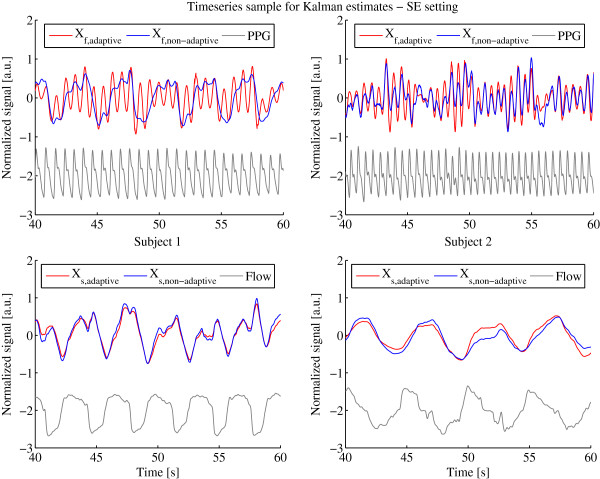
**Internal states*X***_***f***_** and*X***_***s***_** determined by adaptive and non-adaptive Kalman filter with PPG and Flow Reference using the SE setting.** Internal states *X*_*f*_ (top) and *X*_*s*_ (bottom) determined by adaptive (red) and non-adaptive (blue) Kalman filter with PPG and Flow Reference (gray) using the SE setting for subject 1 (left column) and 2 (right column).

**Figure 7:**
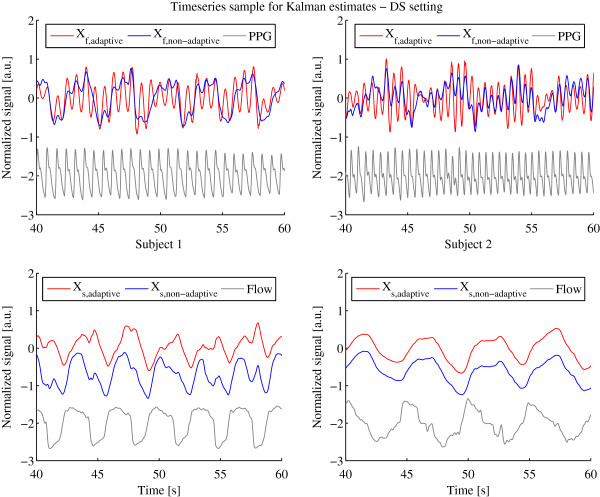
**Internal states*X***_***f***_** and*X***_***s***_** determined by adaptive and non-adaptive Kalman filter with PPG and Flow Reference using the DS setting.** Internal states *X*_*f*_ and *X*_*s*_ determined by adaptive (red) and non-adaptive (blue) Kalman filter with PPG and Flow Reference (gray) using the DS setting for subject 1 (left column) and 2 (right column).

**Figure 8:**
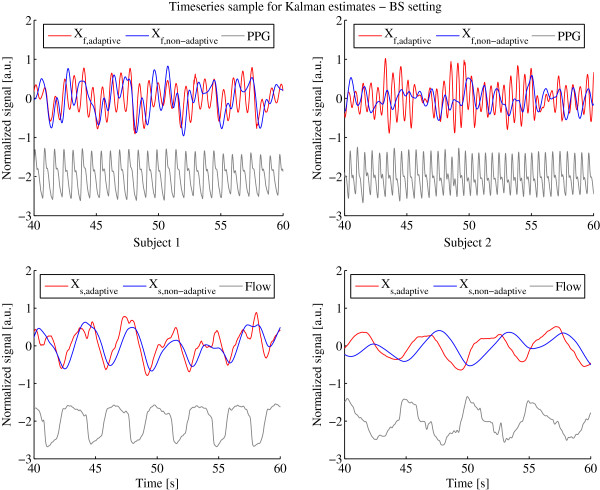
**Internal states*X***_***f***_** and*X***_***s***_** determined by adaptive and non-adaptive Kalman filter with PPG and Flow Reference using the BS setting.** Internal states *X*_*f*_ and *X*_*s*_ determined by adaptive (red) and non-adaptive (blue) Kalman filter with PPG and Flow Reference (gray) using the BS setting for subject 1 (left column) and 2 (right column).

Another performance indicator is the ability to separate the sensor signal into an offset, heart and respiration signals adequately. To evaluate the offset estimation performance we compute the mean error between the offset and the raw sensor signal shown in Table 3. High mean errors imply a high deviation from the original signal and that offsets are not correctly estimated. Note that the error can only be compared within one sensor and subject, since the sensors are not calibrated to the same amplitude range. In all settings most of the mean error and standard deviation values of the adaptive Kalman filter are under or at least very near to the non-adaptive Kalman filter, indicating that it outperforms the non-adaptive filter for offset estimation. To give an insight in the heart and respiration signal separation capabilities we show 20 s of the *X*_*f*_ and *X*_*s*_ values in the middle of the acquired signal in Figure 6, Figure 7 and Figure 8 for the three settings. It is expected that *X*_*f*_ and *X*_*s*_ follow a sinusoidal shaped signal with angular frequencies of *ω*_*f*_ and *ω*_*s*_, respectively. For *X*_*s*_, the signals between the adaptive and non-adaptive Kalman filter resemble through all settings as expected. On the contrary, we see a large amount of respiration on top of the heart rate signal in the non-adaptive *X*_*f*_ which is not present in the *X*_*f*_ of the adaptive filter, regardless of the used configuration. This interaction of *X*_*f*_ with the respiratory signal degrades the performance of detecting the peaks that are needed for heart rate estimations. For BS, the *X*_*f*_ of the non-adaptive filter does not contain any visible heart signal any more, making it impossible to estimate heart rates with the employed peak detection procedure.

**Table 3:** **Mean Error (standard deviation) between estimated offsetC**_**1,k**_- **C**_**3,k**_**and raw sensor signals S1-S3 for the non-adaptive and adaptive (Ad.) Kalman filter**

**Subj.**	**S1-C**_**1,k**_	**S1-C**_**1,k**_**Ad.**	**S2-C**_**2,k**_	**S2-C**_**2,k**_**Ad.**	**S3-C**_**3,k**_	**S3-C**_**3,k**_**Ad.**
**1 (SE)**	16 (4850)	1 (1259)	-0 (644)	11 (571)	-3 (4674)	84 (4027)
**2 (SE)**	-1 (111)	0 (88)	-304 (4016)	-263 (3960)	-429 (5634)	-352 (6028)
**1 (DS)**	-817 (4039)	2 (1253)	-8300 (4172)	21 (574)	-8302 (4119)	171 (4049)
**2 (DS)**	-882 (480)	0 (88)	-8832 (4996)	-246 (3934)	-8832 (4967)	-334 (6009)
**1 (BS)**	861 (11310)	1 (1248)	203 (1264)	8 (569)	-1693 (9014)	53 (4008)
**2 (BS)**	3 (245)	0 (88)	-698 (4239)	-314 (3971)	-498 (6483)	-407 (6033)

To assess whether the described procedure is able to work in real-time, computational time measurements were conducted. The filter procedure was implemented in Matlab R2011a (MathWorks) on a personal computer with a 3.10 GHz dual core processor and 4 GB RAM. In total the adaptive Kalman filter (including all initialization processes and filtering, but excluding reference frequency measurements) in average 35.9 s (24.79% of the total 144.8 s) and 40.5 s (24.80% of the total 163.4 s) processing times are required, respectively for subject 1 and 2. Without adaptation, the procedures take in average 24.2 s (16.73% of the total 144.8 s) and 24.7 s (15.08% of the total 163.4 s). Hence, performing one regular Kalman filter step requires about 1.7 ms out of 10.5 ms at 95 Hz sampling rate. The effective adaptation rate is reduced to 9.5 Hz (every tenth sample equal to 105 ms sampling time) and in average about 50-60% more computational time is needed, i.e. approximately 2.6 ms per sample. Note, that the Kalman filter computations still are at 95 Hz sampling rate. The surplus of 9 ms within the 105 ms window (ten samples) compared to the non-adaptive filter only occurs during the first adaptation sample which takes 1.7 ms+9 ms=10.7 ms in total. This is slightly more than the sampling time of 10.5 ms resulting in a maximum lag of one sample every tenth sample. Therefore we conclude that the intrinsic computations of both filters are real-time compliant for separating respiratory and cardiac signal activity. However, as described in Section ‘Signal extraction and separation’, buffer sizes of 20 s and 10 s are still necessary to estimate breathing and heart rates, respectively. Note that, until now, no special code optimization has been performed, which would further reduce the required computational time and make it possible to integrate the procedures into microcontrollers or digital signal processing units.

Although the signals were acquired on healthy young men in this proof of concept, a target application might be the telemonitoring of elderly at home. The main advantage of this technique is the contactless and unobtrusive way the measurement are performed. The sensors may be arranged in a shirt, a bed or in a chair just to give some examples. Nevertheless, it has to be examined how daily activities (e.g. cooking, vacuuming, mowing the lawn or walking around) affect the performance of detecting breathing and heart rates. The main drawback of the magnetic induction measurement method is that movements relative to the coils cause large artifacts in the signal on top of the desired signal. These may be detected or in a good case even be compensated. We do not expect that the performance is age dependent. Rather the position of the sensors is more crucial for optimal performance. During resting activities (e.g. watching TV, sitting in a chair reading or sleeping) where the number of body movements is small, the developed filter should be able to perform as well as described since it adapts automatically to the measurement conditions.

## Conclusions

The above analysis shows that estimations of respiration and heart rate based on the implemented adaptive Kalman filter perform very well in a real-time acquisition scenario employing contactless sensors measuring cardiorespiratory signals. The direct evaluation of the respiration and heart rate only based on the Kalman filter states and the direct feed-back into the time variant state transition matrix *A*_*k*_ improved the results compared with the non-adaptive procedure. It is also important that the filter does not require a specific signal shape as long as it contains periodic content for respiration and heart activity; therefore, the filter is suitable for all biomedical signals containing two periodic motions on top of large signal offsets. The delays caused by the buffer windows of 20 s for measurement of the respiration rate and of 10 s for the heart rate are necessary to correctly estimate the frequencies with the described peak detection method. The frequency measurement resolution of 0.0175% per bpm inherently generates errors, especially with increased heart rates. For shorter delays and a better frequency resolution, the frequency estimation method needs to be improved, e.g. by up-sampling the signal in the buffer or employing more complex detection algorithms but without neglecting the real-time ability.

In general, this implementation of the adaptive Kalman filter is kept as simple as possible; this allows to port the code to embedded processing units, e.g. microcontrollers or digital signal processors. It is also possible to expand the system to more than three sensors by adding one row and one column in the state transition matrix *A*_*k*_, the system noise co-variance matrix *Q*_*k*_ and the measurement noise co-variance matrix *R*_*k*_ and only one row in the measurement matrix **H**.

Since this analysis is a proof of concept, a larger study with additional subjects needs to be performed to validate all of the above methods.

## Competing interests

The authors declare that they do not have any competing interests.

## Authors’ contributions

DT and JJ developed the employed hardware measurement system and carried out the data acquisition. JF designed the signal analysis and processing with the support of BM and SL, who contributed with their long lasting knowledge in feedback control and automation. All authors carefully revised the manuscript and contributed to the development of the described system and software. All authors read and approved the final manuscript.

## Authors’ information

Jérôme Foussier was born in Cologne, Germany, in 1984. He holds a Dipl.-Ing. degree in Electrical Engineering from RWTH Aachen University, Germany. Currently, he is pursuing the Dr.-Ing. (Ph.D.) degree at the Chair of Medical Information Technology, RWTH Aachen University, where he is also working as a Research Assistant. His research interests include signal processing and classification as well as physiological measurement techniques.

Daniel Teichmann was born in Essen, Germany, in 1982. He holds a Dipl.-Ing. degree in Electrical Engineering from RWTH Aachen University, Germany. Currently, he is pursuing the Dr.-Ing. (Ph.D.) degree at the Chair of Medical Information Technology, RWTH Aachen University, where he is also working as a Research Assistant. His research interests include non-contact monitoring techniques and signal processing.

Jing Jia was born in Shanghai, China, in 1985. She holds a Dipl.-Ing. degree in Electrical Engineering as well as in Economic Science from RWTH Aachen University, Germany. Currently, she is working at ’Philips Medizin Systeme Böblingen GmbH’ in Böblingen, Germany.

Berno J.E. Misgeld received the Dipl.-Ing. (FH) in Electrical and Automation Engineering from University of Applied Sciences, Aachen, Germany, and the M.Sc. degree from Coventry University, Coventry, U.K., in 2003, respectively. In 2007 he received the Dr.-Ing. degree in Biomedical and Control Engineering from Ruhr-University Bochum, Bochum, Germany. From 2006 to 2011 he was a research and development engineer for guidance and flight control systems at Diehl-BGT-Defence, Ueberlingen, Germany. Since 2011 he is a Senior Scientific Engineer in Biomechatronical Systems and Rehabilitation Robotics at the Chair of Medical Information Technology at RWTH Aachen University, Aachen, Germany. His research interests include feedback control and filtering with application to biomedical systems, robotics and medicine.

Steffen Leonhardt was born in Frankfurt, Germany, in 1961. He holds an M.S. in Computer Engineering from SUNY at Buffalo, NY, USA, a Dipl.-Ing. in Electrical Engineering and a Dr.-Ing. degree in Control Engineering from the Technische Universität Darmstadt, Germany, and a M.D. in Medicine from J. W. Goethe University, Frankfurt, Germany. He has 5 years of R&D management experience in medical engineering industry and was appointed Full Professor and Head of the Philips endowed Chair of Medical Information Technology at RWTH Aachen University, Germany, in 2003. His research interests include physiological measurement techniques, personal health care systems and feedback control systems in medicine.

## Pre-publication history

The pre-publication history for this paper can be accessed here:

http://www.biomedcentral.com/1472-6947/14/37/prepub
